# Evolution of Genomic Base Composition: From Single Cell Microbes to Multicellular Animals

**DOI:** 10.1016/j.csbj.2019.03.001

**Published:** 2019-03-07

**Authors:** Jon Bohlin, John H.-O. Pettersson

**Affiliations:** a Norwegian Institute of Public Health, Division of Infection Control and Environmental Health, Department of Infectious Disease Epidemiology and Modelling, Lovisenberggata 8, 0456 Oslo, Norway; b Marie Bashir Institute for Infectious Diseases and Biosecurity, Charles Perkins Centre, School of Life and Environmental Sciences and Sydney Medical School the University of Sydney, New South Wales 2006, Australia; c Zoonosis Science Center, Department of Medical Biochemistry and Microbiology, Uppsala University, Uppsala, Sweden; d Public Health Agency of Sweden, Nobels vg 18, SE-171 82 Solna, Sweden; e Centre for Fertility and Health, Norwegian Institute of Public Health, PO-Box 222 Skøyen, N-0213 Oslo, Norway; f Norwegian University of Life Sciences, Faculty of Veterinary Sciences, Production Animal Clinical Sciences, Ullevålsveien 72, 0454 Oslo, Norway

## Abstract

Whole genome sequencing (WGS) of thousands of microbial genomes has provided considerable insight into evolutionary mechanisms in the microbial world. While substantially fewer eukaryotic genomes are available for analyses the number is rapidly increasing. This mini-review summarizes broadly evolutionary dynamics of base composition in the different domains of life from the perspective of prokaryotes. Common and different evolutionary mechanisms influencing genomic base composition in eukaryotes and prokaryotes are discussed. The conclusion from the data currently available suggests that while there are similarities there are also striking differences in how genomic base composition has evolved within prokaryotes and eukaryotes. For instance, homologous recombination appears to increase GC content locally in eukaryotes due to a non-selective process termed GC-biased gene conversion (gBGC). For prokaryotes on the other hand, increase in genomic GC content seems to be driven by the environment and selection. We find that similar phenomena observed for some organisms in each respective domain may be caused by very different mechanisms: while gBGC and recombination rates appear to explain the negative correlation between GC3 (GC content based on the third codon nucleotides) and genome size in some eukaryotes uptake of AT rich DNA sequences is the main reason for a similar negative correlation observed in prokaryotes. We provide further examples that indicate that base composition in prokaryotes and eukaryotes have evolved under very different constraints.

## 1 Introduction

In prokaryotes and eukaryotes, the genome consists of one or several DNA molecules that contain the genetic information of the organism. While all prokaryotes and eukaryotes have genomes consisting exclusively of DNA molecules some viruses have, in addition to single and double stranded DNA genomes, RNA genomes that are also either single or double stranded [1]. The DNA molecule is stable due to the double stranding resulting from the coupling of adenine (A) to thymine (T) and guanine (G) to cytosine (C) (or vice versa) [2]. Genomes are dynamic in the sense that offspring evolve through mutation, or recombination. More specifically, genomes change either due to mutation of bases (e.g. C changes to T during replication), loss of long or short stretches of nucleotides (including genes), replication of (oligo-) nucleotides, rearrangements due to for instance, transposons, recombination, duplication, transformation, conjugation and/or transduction [2,3].

### 1.1 Genomes in the Three Domains of Life

The structure of an organisms' genome varies according to the domain of life it belongs to. Prokaryotes, which include both the domains of bacteria and archaea, have small and highly energy-efficient genomes, typically consisting of a few million base-pairs [4]. The base composition in the genomes of archaea and bacteria can vary quite substantially between different species although genes and proteins may be similar or even identical [5]. Organisms from the two domains also share genomes with a large fraction coding for proteins [2]. Eukaryotes, on the other hand, have genomes ranging from the very small, approximately that of the larger bacteria (i.e. Encephalitozoon cuniculi with its 2.9 Mb genome [6]), to the very large consisting of over a 100 billion nucleotides [7], such as the lungfish Protopterus aethiopicus (130 billion base pairs [bp]) [8] and the monocot plant Paris japonica (150 billion bp) [9]. Some amoebas have even larger genomes; Amoeba dubia has a genome with an estimated size of 670 Gbp [10]. Genomic base composition variation is typically less between eukaryotic species than between prokaryotic species. However, base composition varies less within prokaryotic genomes than within eukaryotic genomes.

### 1.2 Virus and Phages

Since viruses are classified as neither eukaryotes nor prokaryotes, they will only be discussed briefly in the current section of this review. For factors and determinants driving evolutionary change in viruses see, for instance, [[11], [12], [13], [14]]. Viruses are taxonomically classified into groups I–VII depending on the genome type (i.e. segmented/non-segmented, single/double stranded RNA/DNA, positive/negative sense) and particular groups show distinct affinity towards each respective domain [1]. Viruses exclusively associated with archaea and bacteria are commonly referred to as phages [1,3,15]. Phages have primarily single or double stranded DNA based genomes (designated Group II and Group I, respectively) that are on average smaller than those of viruses infecting eukaryotes [3]. Archaeal and bacterial phages appear to be largely exclusive to each respective domain and overlap seems to occur only rarely [16]. The viral genomes associated with eukaryotic hosts may consist of single stranded RNA, such as *Ebolavirus* (Group V) and HIV (Group VI), double stranded RNA (*Rotavirus*, Group III), single stranded DNA (*Parvovirus*, Group II), or double stranded DNA (*Adenovirus*, Group I) [1]. Phages appear to mimic the base composition of their bacterial hosts closely, viruses associated with eukaryotic hosts less so [17]. Unlike phages, viruses with eukaryotic hosts can have genes consisting of exons and introns [3]. Although phages often have similar base composition to that of their hosts the genomes are almost always (slightly) more AT-rich [18]. Viral genomes tend to be small with a high fraction of genes, but the largest viruses, such as the *Pandoravirus* (Group I), with its 2.5 Mb sized genome, is comparable to that of smaller prokaryotes [19].

### 1.3 Base Composition, Genome Size and Ploidy

**Fig. 1:**
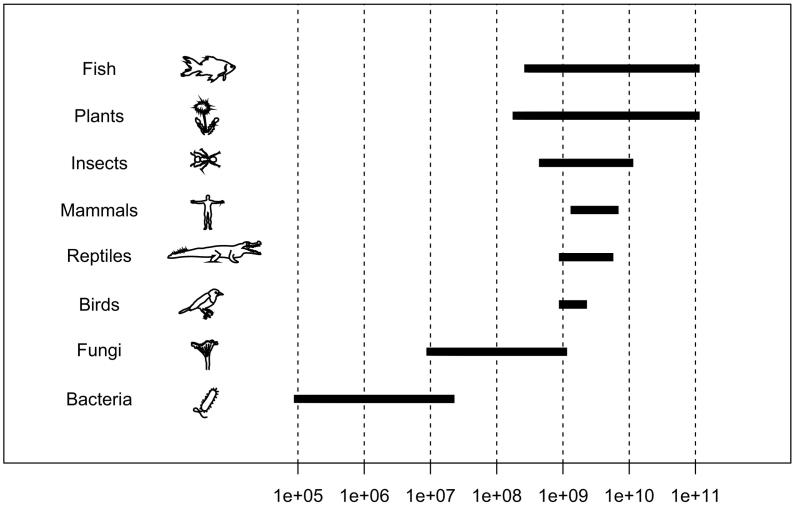
Genome sizes in selected organisms. The figure shows approximate genome size range in log scale bp (horizontal axis) for a diverse set of organisms (vertical axis).

Base composition is generally more similar within closely related groups and organisms residing in the same environments [3,[20], [21], [22]]. For instance, the average genomic GC content of the currently sequenced avian, mammalian and reptilian genomes all lie somewhere within 40–50%GC [23,24], with GC3 (GC content of nucleotides in third codon position) slightly higher [25]. GC content in bacteria and archaea range from approximately 13–75%GC [24] and genomic %GC correlates strongly with GC3 [26]. Whereas the genome size of mammals is somewhat larger than that of reptiles and birds (roughly 3 Gb vs 2 Gb) [7,27] they have far from the largest genomes; several plants [28] and fishes (e.g. bread wheat 17 Gb and lungfish 150 Gb, respectively) have substantially larger genomes with more protein coding genes than mammalian genomes [29,30]. That genome size does not increase with the complexity of the organism is known as the C-value paradox [31] (See Fig. 1). The karyotypes and ploidy (the sets of homologous chromosomes in a genome) can also vary, not only in plants and animals, but also in prokaryotes [32]. The extremely radiation-resistant *Deinococcus radiodurans* can have as many as 10 copies of its two chromosomes [33]. The chromosomes of most bacteria and archaea, however, are single copies but many bacteria have plasmids, which replicate independently of chromosomes [34]. They are often present in large copy numbers which can be advantageous for avoiding antimicrobial treatment [3,35]. Some prokaryotes have genomes consisting of two and even three independent non-homologous chromosomes (for instance *Burkholderia cenocepacia* [36]), but the norm is one chromosome in both archaeal and bacterial genomes [2]. In eukaryotes, on the other hand, most organisms have multiple chromosomes of which the number varies substantially even between closely related species and genera [37]. Chromosomes undergoing fusion (or fission from one chromosome into several) may be a driver for genetic variance in eukaryotes as it may break linkage and therefore lead to substantially more phenotypic variation than single mutations [38]. Phenotypic changes correlate with genotypic changes, but mutations and genomic re-arrangements do not necessarily result in phenotypic changes. In contrast to eukaryotes, prokaryotes have evolved highly optimized genomic systems with advanced mechanisms for both DNA gain and loss [3]. Moreover, approximately 90% of bacterial genomes consist of genes [2,3] compared to 1–2% in mammals [39,40]. In addition to prokaryotes' highly efficient DNA housekeeping systems [3], the large fraction of gene-coding DNA is most likely also related to prokaryotes' relatively short doubling time. Some bacteria may double as fast as in a few minutes (e.g. *Bacillus cereus* and *Vibrio cholerae*) [2] implying that populations may expand substantially in size within just a few hours. Larger multicellular animals require years to produce offspring and therefore population sizes are very small compared to that of bacteria [32]. Many evolutionary mechanisms relating to genomic base composition in both eukaryotes and prokaryotes are more practical to study from the viewpoint of prokaryotic genomes since thousands of genomes are publically available for scrutiny. In other words, we know considerably more about prokaryotic genomics than eukaryotic genomics and therefore the point of this mini-review is to explore the evolution of genomic base composition in both domains but through the lens of prokaryotic genomics.

## 2 Base Composition in Prokaryotes

### 2.1 Chargaff's Parity Rules

Chargaff's first parity rule states that purines (A/G) and pyrimidines (C/T) occur in approximately similar frequencies within a double stranded DNA genome [30]. Furthermore, Chargaff's second parity rule says that respectively A/T and G/C bases occur in similar frequencies on each strand [41]. While these rules appear to be valid for all organisms with genomes consisting of double stranded DNA deviations may occur in viral genomes consisting of single stranded RNA or DNA [42]. Chargaff's parity rules may also be valid for short oligonucleotides and their reverse complements, at least up to a certain size [43,44]. But, again, predominantly in organisms with genomes consisting of double stranded DNA [41]. It has been suggested that Chargaff's parity rules may be a consequence of repeated inversions and inverted transpositions during the course of evolution [41], but the issue is still debated [45].

### 2.2 Genome Evolution within and between Prokaryotes

While Chargaff's parity rules appear to be applicable within genomes with double stranded DNA GC content can vary greatly between prokaryotic genomes [2,22]. There can also be substantial regional %GC differences within genomes but these are typically limited to shorter regions (i.e. seldom more than thousands of Kb) [46]. While some bacterial phyla, such as the Firmicutes, are predominantly AT rich others, like Actinobacteria, are mainly GC rich [2,4]. Most prokaryotic phyla, however, consist of species with varying genomic %GC [2]. One of the most AT rich bacterium sequenced is the β-proteobacterium *Candidatus* Zinderia insecticola with 82.5% AT [47]. The size of this bacterium's genome is among the smallest consisting of only 208,564 bp. The genome of *Candidatus* Tremblaya princeps (also β-proteobacteria) is even smaller with 138,927 bp but with an average genomic GC content of 58.8% [48]. One of the largest genomes belongs to the δ-proteobacterium *Sorangium cellulosum* and consists of 13,033,779 bp of which 71.4% is GC [49]. It is not known exactly why genomic base composition vary so much between different bacteria, even among those from the same phylogenetic group, and why it differs so little within. The data from whole genome sequencing provides several clues. For instance, GC rich bacteria appear to be large and soil-dwelling with more complex genomes as opposed to AT rich bacteria which are often intra-cellular symbionts, or parasites, with small genomes [2,3]. Why microbes become more AT rich appears to be easier to explain than why prokaryotic genomes increase in %GC; it has been convincingly argued that relaxed selection can lead to loss of DNA repair genes which in turn lead to the accumulation of AT-rich mutations [50] due to the now established C → T mutation bias [4,51]. In fact, it has been shown that GC → AT mutations occur approximately twice as often as AT→GC mutations, which seems to be more readily fixed within the genome [51,52]. Many bacteria subjected to relaxed selection are often intra-cellular, living in low density [53] populations with little chance of recombining or exchanging DNA with other bacteria [47]. Mutations are therefore not necessarily purged from genetic regions that are not of vital importance to the organism resulting in both increased AT content, number of defective genes (pseudogenes), but also novel proteins [54]. Microbial genomes with increased %GC, on the other hand, can be a consequence of the fact that nitrogen is often abundant in soil [55]. It could also be a trade-off between energetically expensive nucleotides for cheap amino acids [56]. Indeed, G binds to C with three hydrogen bonds, as compared with two for A and T, implying that base-stacking is, in general, more energetically expensive for guanine and cytosine nucleotides [2]. Soil bacteria are often more GC rich, have larger genomes and more complex gene regulation [57] than host associated bacteria therefore it is interesting to note that genome size correlates with GC richness in Proteobacteria and Actinobacteria [58,59], and possibly in other phyla as well. For more closely related bacteria, i.e. strains of species, a negative correlation has been observed between genome size and GC content [59]. This is most likely due to the incorporation of AT rich foreign genetic elements into the host chromosome [59,60]. Indeed, most foreign genetic elements, such as phages and plasmids, are more AT rich than the host chromosome [61,62]. Microbial accessory genomes have also been found to be, on average, slightly more AT rich than the more conserved core genomes and it appears to be preservation of the core genome through purifying selection that is responsible [61]. While genes belonging to the accessory part of the genome may have been widely transferred among other strains, and even species or genera, core genomes have, after all, been retained in a number of strains and are therefore, most likely, crucial to the respective species [61,63]. Phylogenetic relatedness is also a factor determining base composition in microbes but it seems to be limited primarily to the species and genus level [20,61]. Genomic changes can however occur fast in microbes; even those closely related do not necessarily share the same preferences for codons [64,65]. Some have suggested that codon preference may be due to the presence of particular tRNA genes, but evidence is mounting that genomic GC content, and to some extent phyla [66], are the driving factors [67]. If so, it could imply that while codon preference is determined to some degree by phylogeny environmental influences, mediated by selective pressures or lack thereof, can too exert substantial influence [20]. Coding regions (excepting RNA-genes) are in general significantly more GC rich within a genome than non-coding regions [68].

There have been some proposals that recombined genetic regions are more GC rich than expected and that this could be due to a selective neutral process [69,70]. This process has been termed GC-biased gene conversion (gBGC) and may be a consequence of DNA repair integrating recombined stretches of DNA into the host chromosome by predominantly filling in the energetically more expensive but more robust G/C nucleotides [56,70]. There appears to be statistical evidence supporting increased %GC in heavily recombined regions, and gBGC, in eukaryotes [69] but disentangling gBGC from purifying selection in the more fast replicating and larger populations of prokaryotes appears to be challenging [20,61,71]. While it has, in fact, been observed that the core genomes of several intra-cellular, seldom recombining, symbionts or parasites are just as AT-rich as the corresponding accessory genomes [61], reinforcing the assumption of gBGC-like mechanism in prokaryotes, statistical associations may unfortunately say little about actual causation. Indeed, intracellular symbionts/parasites often lack DNA repair genes and inhabit environments with relaxed selective pressures, including purifying selection, which could just as well explain the similar GC-content observed both for core- and accessory genomes [47,72].

### 2.3 Strand-Biased Base Composition

**Fig. 2:**
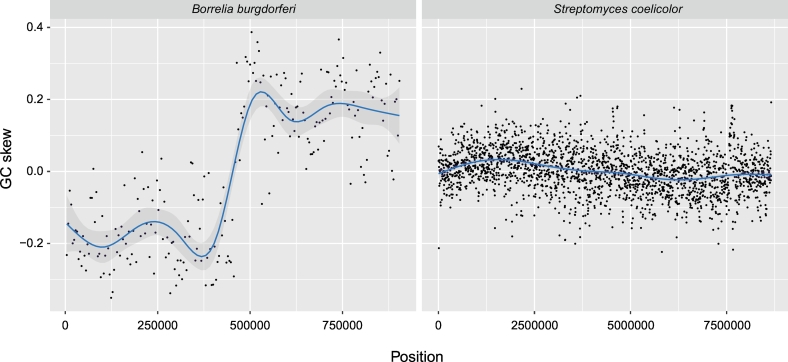
GC skew in two bacteria with linear chromosomes. The figure demonstrates GC skew in two bacteria with linear chromosomes using a 4 Kb sliding window. The horizontal axis designates chromosome position while the vertical axis denotes the GC skew. The left panel shows the GC skew for the intra-cellular pathogen *B. burgdorferi* while the panel to the right displays the GC skew for the soil bacterium *S. coelicolor*.

In many prokaryotes [73], and in some eukaryotes [[74], [75], [76]], it can be seen that the occurrence of G's is substantially more pronounced on the leading strand than C's [4,77,78]. The phenomenon is commonly referred to as GC skew (see Fig. 2). To a lesser extent it can also be observed that T's are more common on the leading strand than A's [77]. On the lagging strand the occurrence of G's and C's is reversed as well as T's and A's. It is therefore possible to use the GC skew to predict the origin of replication [79]; it can be found at the crossing point where G's (and to a lesser extent T's) become more frequent on the leading strand and C's (and A's) on the lagging strand [4,77]. There also exists bacteria with excessive C's on the leading strand, such as the large soil bacterium *Streptomyces coelicolor*, and many bacteria do not exhibit any such coherent skew at all therefore some care must be taken when determining the origin of replication using only GC skews in unfamiliar prokaryotes [80]. Some bacteria, like *Bacteroides thetaiotaomicron*, also have multiple origins of replication which can also be observed from the GC skew [80]. Although GC skews in bacteria have been known for decades a satisfactory unifying explanation is still not available. There are several suggestions [41,80,81] and therefore it is still a debated issue. Fast replicators, such as *B. cereus*, appear to have more pronounced skews than slow replicators, such as *Mycoplasma hyopneumoniae* [80]. It could be an association between GC skew and optimal growth temperature, but also this is difficult to prove or explain [2]. Whether the genome of the bacterium is linear or circular does not seem to be of importance as *Borrelia burgdorferri* has a pronounced GC skew while *S. coelicolor* does not [4,80]. Some progress have been made in explaining nucleotide skews in the Firmicutes, more specifically in *Staphylococcus aureus*, and all evidence points towards selection since it is primarily the amino acid changing nucleotides in each codon that appears to be affected [82]. Many Firmicutes are also fast growers and such microbes tend to have considerably more genes on the leading strand than on the lagging strand [83]. The Firmicutes, however, seems to differ from many other prokaryotic phyla with regards to GC skew since A's are more abundant on the leading strand (and T's on the lagging strain) [82]. Although not all prokaryotes have pronounced GC skews other nucleotide patterns often exist that can be used to identify the origin of replication, even in slow growing bacteria. Indeed, examination of oligonucleotide skews up to heptamers has proved to be a successful method in determining the origin of replication in many microbial species, including slow growing microbes without a pronounced GC skew [79,80]. What forces are responsible for these oligonucleotide skews however remains no more comprehensible than the nucleotide-based skews. The lagging strand is differently assembled than the leading strand by Okazaki fragments, and that, as well as the direction of the genes transcribed, could potentially influence how nucleotides are distributed among leading and lagging strands [80,84,85]. Recently acquired and integrated DNA, from the environment or other foreign sources, will typically affect nucleotide skews as the base composition of such DNA, having been subjected to different selective pressures, is often substantially different to that of the host chromosome [84]. During the course of time however, the base composition of integrated foreign DNA tends to ameliorate towards that of the host chromosome and will eventually attain similar base composition patterns and skews to that of the neighbouring regions [81,86]. Finally, it is tempting to speculate that the observed skews in nucleotide composition has something to do with both of the above-mentioned Chargaff's parity rules as purines and pyrimidines are evenly distributed on both strands and A/T as well as G/C respectively occur in similar frequencies on each strand. The effectiveness of genomic oligomer frequency skews to predict the origin of replication could also be an indirect consequence of Chargaff's second parity rule [41,44].

In summary, base composition in prokaryotic genomes can be seen as a consequence of environment, taxonomic relatedness, availability of essential compounds, selective pressures, population structure, doubling time, transfer of DNA, genome size and more. The challenge laying ahead is to determine the proportional influence from each of these factors.

## 3 Base Composition in Eukaryotes

**Fig. 3:**
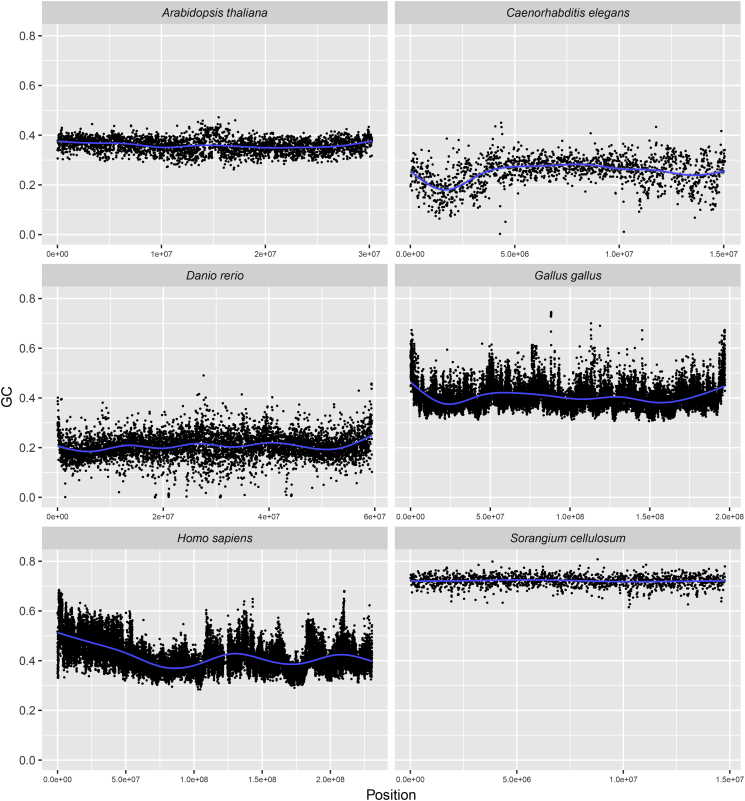
Genomic GC content difference in eukaryotes and prokaryotes. The figure demonstrates GC contents (vertical axis) of chromosome 1 in the eukaryotes thale cress (*A. thaliana*), roundworm (*C. elegans*), zebrafish (*D. rerio*), chicken (*G. gallus*), human (*H. sapiens*), and the prokaryote *S. cellulosum* using a 10 Kb sliding window. The horizontal axis denotes position (bp).

In contrast to eukaryotes, bacteria can quickly replicate into very large populations. The large number of individuals in microbial populations allows for both streamlining and purifying selection to operate at considerable higher rates than that observed for eukaryotes [53]. Indeed, the most abundant bacterium on earth *Candidatus* Pelagibacter ubique, found in all large oceans, has a median intergenic spacer size of only 3 bp per gene (i.e. approximately 96% of the genome codes for genes), very few pseudogenes and a surprisingly small genome (1.3 Mb) for a free-living bacterium [87]. *Candidatus* Pelagibacter ubique has a highly streamlined genome and a doubling time of approximately 30 min [87]. Eukaryotes with large bodies like birds and mammals may in contrast require several years to produce a single progeny [23,88]. Populations of large-bodied animals are therefore small, which implies that streamlining and purifying selection will require much more time to operate effectively on their genomes. The large percentage of non-coding DNA in eukaryotes could therefore be a consequence of the time it takes purifying selection to purge the vast amounts of non-coding DNA cumulating in slowly replicating organisms. Similar mechanisms have, in fact, been observed for the genomes of several bacteria typically moving from one environmental niche to another. More specifically, it can be seen from *Sodalis glossinidus* [89] and *Mycobacterium leprae* [90,91], both having in recent times entered into a obligate intracellular life style, that the fraction of non-coding DNA (approximately 50% and 70% for each species, respectively) has increased considerably compared to their closest relatives. The relatively large number of pseudogenes in these bacteria's genomes could be due to a lack of time having passed for the non-functional DNA to be lost. In a similarly manner, it can be conceived that the genomes of eukaryotes may contain DNA that has simply not been lost due to the long reproduction times and, at least compared to prokaryotes, small populations [92]. Since eukaryotic cells are in general very different from prokaryotic cells selection upon genomic base composition would most likely operate in a different manner as compared to that of prokaryotes [3]. Indeed, mitochondria (and additional plastids in plants), which are present in most eukaryotic genomes, provide extra energy for the cell that could reduce the selective pressure on, for instance, genome size resulting in the accumulation of large chunks of non-coding DNA [93]. Sexual reproduction, which is exclusive to eukaryotes, is also bound to affect genome structure through recombination and preservation of genetic regions [3]. Large contiguous regions (typically several 100's of Kb's) within eukaryotic genomes have been found to have remarkably similar base composition [94]. These homogeneous regions, in terms of GC content, of genomic DNA have been termed isochores and are characterized as mosaic genomic fragments [94,95]. While isochore-theory is debated [96] the heterogenic GC content regions have been observed, although to varying degrees, within many eukaryotic species (See, for instance, Fig. 3) [23,[94], [95], [96], [97], [98]]. Genomic structures such as isochores have not been identified in prokaryotes and therefore seem to represent a layer of structural chromosome organization distinct to eukaryotes [98]. Indeed, isochore-like structures have been linked to chromosomal packaging, in nucleosome-dense regions, and higher order chromosome structure [97]. Since eukaryotic genomes are considerably larger than prokaryotic genomes isochore-like structures may have evolved as a necessity to organize the large chromosomes in eukaryotes [99]. Although isochore-like structures are not reported in prokaryotic genomes, there seems to be indications that some prokaryotic chromosomes can also be organized into higher order structures not unlike that of eukaryotes [100,101]. Chromatin structure, which is responsible for the higher order structuring of DNA in eukaryotes with no counterpart in bacteria and only rudimentary variants in Archaea [102], may have been an important factor influencing homogeneous genetic regions such as CpG islands and shores as well as repetitive regions and thereby the formation of isochore-like structures [97,98].

Another genomic similarity observed in some eukaryotic and prokaryotic species is the negative correlation between genome size and GC3 [25,27,59]. As was explained above, while some closely related prokaryotes have negatively correlated genomic GC content with genome size, predominantly due to uptake of AT rich fragments of DNA [43], the same phenomenon observed in some eukaryotes may [31], on the other hand, be due to quite different mechanisms. Body size has for some birds and reptiles been found to correlate with genome size [25,27,88]. Large animals tend to live longer and therefore producing offspring more seldom resulting in slightly less optimized genomes due to relatively low population sizes [92]. Since GC content correlate negatively with body size, as well as chromosome size, it is assumed that the genomes of animals with smaller genomes that have reproduced more frequently have been subjected to more homologous recombination and therefore also more effective gBGC, resulting in more GC rich GC3 nucleotides [23,25,88]. Hence, while uptake of foreign AT rich DNA seems to explain the majority of the negative associations between GC content and genome size in bacteria, body size and chromosome size together with gBGC could be the driving cause for the same phenomenon observed in some mammals, birds and reptiles [23,25,31]. Due to the small populations and slow reproduction times gBGC may have evolved in eukaryotes as a necessary mechanism to counter the AT rich mutation bias.

## 4 Structural Differences Between Eukaryotic and Prokaryotic Chromosomes

### 4.1 Gene Structure

Prokaryotic genes are often seen as linear continuous stretches of DNA coding for proteins or RNA. This picture is somewhat not representative for eukaryotes since genes are divided into regions called exons and introns [3]. In other words, gene finders made for prokaryotes are not of much use with eukaryotes [103]. It has been argued that introns are most likely non-purged mobile genetic elements, like IS sequences, that can be traced all the way back to prokaryotes [3]. Some appear not to have any functions while other may have evolved, maybe as a consequence of exaptation [99,104], to facilitate multiple gene variants in eukaryotes, also known as alternative splicing [99]. While mobile genetic elements and transposons are common to both prokaryotes and eukaryotes, introns seem to be particular to eukaryotes [3].

### 4.2 Chromosome Structure and Karyotypes

Most bacterial chromosomes are circular, although some do have both linear chromosomes and linear plasmids [4]. The causative agent for Lyme's disease, *B. burgdorferrii*, is one example of such a bacterium [105]. The genomes of eukaryotes are predominantly divided into multiple linear chromosomes [32,106]. Small repetitive stretches of DNA are attached to each end of the chromosome. These small sequences of repetitive DNA are called telomeres and contract during the course of repeated chromosome replication in some cell types [106]. This happens during mitosis in eukaryotes and that, most likely, describes the negative correlation between telomere length and the age of an organism in certain cell types [106,107]. Bacteria with linear chromosomes do not have telomeres and the ends of the chromosome are typically wrapped up by closed hairpin-loop ends [108].

Genomic DNA in most eukaryotes is wrapped 1.65 times (147 bp) around octamer histone protein complexes collectively called nucleosomes [109,110]. Regions of DNA, containing several nucleosomes are, in turn, wrapped around other proteins organizing DNA into an additional structural layer [110]. These resulting chromosomal structures are once again wrapped around the previous structures resulting in an even higher order of organization [110]. The number of levels of the chromatin structure can vary between organisms such as plants and animals [109]. Not only does this hierarchical multi-level organization of eukaryotic chromosomes facilitates storage of large sequences of DNA into less space but it also provides mechanisms for gene regulation as changes to chromatin structure has profound effect on gene expression [111]. Chromatin changes appear as fundamental structural differences, at least seen from a microscope, and activity is most pronounced during mitosis [111]. It is tempting to speculate that chromatin structure may have evolved as a consequence of the larger genomes found in eukaryotes. Chromatin structures have however also been identified in eukaryotes with small genomes such as *Schizosaccharomyces pombe.* The genome size of *S. pombe* is approximately 14 Mb, which is close to that of large bacteria (i.e. the soil bacterium *S. cellulosium* has a genome size of 13 Mb) [112]. Furthermore, since chromatin organization is also strongly coupled with gene regulation [110] there can be many factors responsible for chromatin evolution and maintenance. Nevertheless, GC-rich regions tend to be nucleosome depleted [113], possibly due to the stiffening effect of GC-rich sequences [114]. While GC-rich sequences may be more rigid, no negative correlation was observed in a recent study [115] between local GC content and mutation rates. Rather, it was found that intrinsic DNA curvature was negatively correlated with mutation rates, i.e. increase in curvature leads to lower mutation rates, which could suggest low mutation rates in nucleosome-dense regions.

### 4.3 The Different Paths of Base Composition Evolution in Eukaryotes and Prokaryotes

Microbial genomes have very optimized genomes with respect to energy economy. In practice, pseudogenes and non-functional DNA tend to be lost quickly in prokaryotes [47]. However, change of environment, with a corresponding alteration in selective pressures, can result in accumulation of pseudogenes and/or other types of non-functional DNA [89]. There seems to be a drive towards constantly minimizing superfluous DNA and hence a selection for ‘economic’ energy expenditure [93]. Furthermore, as can be seen from Fig. 3, nucleotide patterns are very similar throughout prokaryotic genomes, excluding plasmids [46]. Compartmentalization of heterogenic genetic regions with similar GC content, such as those described above for isochores, are not seen in prokaryotes, except for GC content differences due to uptake of foreign DNA [116]. The isochore-like structure of compartmentalized regions with similar GC content has been suggested to be partly a consequence of gBGC due to recombination [23]. In prokaryotes a negative correlation between genome size and GC content (that correlate with GC3) was found to be due to uptake of foreign genetic regions and was not related to homologous recombination and gBGC as seems to be the explanation for the eukaryotic genomes in question [18,59,117]. Hence, the data available may suggest that the evolution of base composition in eukaryotic genomes could be associated with cross-over recombination rates and gBGC [23,25,[118], [119], [120]]. In prokaryotes, on the other hand, the evolution of base composition appears to be more directly linked to life style and associated selective pressures [20,71,72]. Thus, from the scant genomic data currently available for eukaryotes the gBGC hypothesis suggested for prokaryotes [70] does not seem very convincing as others have already pointed out [71]. The highly efficient and low fraction of non-coding DNA found in prokaryotic genomes makes the hypothesis of economizing energy expenditure a very compelling argument for the differences in genome sizes between prokaryotes and eukaryotes [93,121]. Indeed, the small parasitic eukaryote *E. cuniculi* with a genome the size of an average bacterium, described above, also lacks mitochondria [6]. In addition, genome duplication is as of yet only documented in eukaryotes [122]. Due perhaps to mitochondria and plastids, eukaryotes do not appear to have the same drive to minimize energy expenditure through, for instance, removal of non-functional DNA [121,[123], [124], [125]]. As was recently pointed out by Eugene Koonin, selective pressures act differently on genome size in eukaryotes, as compared to prokaryotes, leading instead to specialized genomic inventions, not found in prokaryotes, possibly due to exaptation resulting in different systems for gene regulation [99]. If so, genomic evolution in eukaryotes can have taken a very different route than what has currently been observed for prokaryotes [126]; while base composition evolution in prokaryotes is tightly associated with natural selection mediated by the environment, selective neutral processes, such as gBGC, linked to cross-over recombination could be one mechanism moulding base composition in eukaryotes.

## 5 Summary and Outlook

We have reviewed current research with a particular focus on base composition evolution in both prokaryotes and eukaryotes but from the perspective of microbial genomics. Our findings suggest that there are substantial differences between eukaryotic and prokaryotic genomes. For instance, we find particular to eukaryotes genomic GC content increase notably in regions subjected to frequent recombination. This is not observed to the same extent in prokaryotic genomes. A negative correlation between genome size and GC3 has been observed in some eukaryotic species and this is presumed to be related to recombination. Indeed, for many of the same eukaryotes, the correlation between GC3, body size, and longevity, factors that have all been associated with recombination rates, suggests that gBGC may be the source. A similar negative correlation between genomic GC content and genome size for the strains of several prokaryotic species on the other hand points to uptake of foreign DNA, which is often more AT rich than the host genome.

The eukaryotes have, on average, much larger genomes than prokaryotes, with only a small fraction coding for genes, in contrast to the large fractions of coding DNA found in prokaryotes. The large genomes of eukaryotes could be a consequence of low population densities and long life cycles, in contrast to prokaryotes, but it could also be a result of increased cellular availability of adenosine triphosphate (ATP) due to plastids and mitochondria as abundance of energy could reduce selective pressures for smaller genomes. The findings here will most likely be supplanted in the near future, especially what has been described regarding eukaryotic genomes, as there is still a scarcity of such genomes available for analysis. Furthermore, most eukaryotic genomes available are only available as drafts and therefore not completely assembled and closed as is the case for thousands of prokaryotic genomes.

## 6 Materials and Methods

The genome size range in Fig. 1 was obtained from the animal Genome Size Database [8] and the figure was made using the statistical package R [127]. The GC content and GC skew of the organisms depicted in Fig. 2, Fig. 3 were computed using in-house scripts and the figure was made using the ggplot2 library [128] and R.

Chromosome 1 from the eukaryotes depicted in Fig. 3 had the following accession numbers: NC 003070.9 (*A. thaliana*), NC 003279.8 (*C. elegans*), NC 007112.7 (*D. rerio*), NC 006088.5 (*G. gallus*), NC 000001.11 (*H. sapiens*). The accession number of the prokaryotes in Fig. 2, Fig. 3 were: NC 021658 (*S. cellulosum*), NC 001318 (*B. burgdorferi*) and NC 003888 (*S. coelicolor*). All genetic data was downloaded from NCBI [129].

## Declarations of Interest

None.
